# Epidemiological and clinicopathological analysis of basal cell carcinoma in Egyptian population: a 5-year retrospective multicenter study

**DOI:** 10.1007/s00432-022-04207-7

**Published:** 2022-07-23

**Authors:** Mohamed El-Khalawany, Hussein M. M. Hassab-El-Naby, Ahmed Mustafa Mousa, Ahmed Sameh, Mahmoud A. Rageh, Rasha Mahmoud Genedy, Aya Magdy Hosny, Marwa A. Aboelmagd, Soha Aboeldahab

**Affiliations:** 1 Department of Dermatology, Faculty of Medicine, Al-Azhar University, Cairo, Egypt; 2 Department of Dermatology, Egyptian Armed Forces College of Medicine (AFCM), Cairo, Egypt; 3 Department of Dermatology, Faculty of Medicine, Alexandria University, Alexandria, Egypt; 4 Department of Dermatology, Faculty of Medicine, Sohag University, Sohag, Egypt

**Keywords:** Basal cell carcinoma, BCC, Keratinocyte carcinoma, Epidemiology, Skin cancer, Non-melanoma skin cancer, Clinical dermatology, Dermatopathology

## Abstract

**Purpose:**

Basal cell carcinoma (BCC) is the most diagnosed type of cancer accounting for 80% of all keratinocyte malignancies. However, the exact demographic properties and clinicopathological criteria for BCC in Egyptians are not clearly reported. Our aim is to report and analyze the epidemiological and clinicopathological features of BCC in Egyptians.

**Methods:**

We retrospectively reviewed the medical records for patients diagnosed pathologically with BCC during the period from January 2017 to December 2021. Data were recruited from four dermatology centers with different geographical distributions.

**Results:**

We registered 544 patients. Their age ranged between 22–91 years with a mean of 61.6 ± 13.2 years. Females showed younger age of onset. The mean duration of the tumor was 3.9 ± 3.8 years. The most common involved region was the head (79.4%), and about one third of patients (32.2%) had a giant lesion (> 5 cm). The most common clinical presentation was ulcerative lesions (44.9%). Pathologically, the nodular type represented the most common variant (50.4%).

**Conclusion:**

Our results proposed that the annual incidence of BCC is increasing among Egyptians. Ultraviolet radiation is considered a high-risk factor of BCC leading to a higher affection of the head region and more prevalence in men. This study also highlights some criteria of BCC in Egyptians such as the long duration of the tumor, the early onset in females, the higher percentage of giant types, and the predominance of nodular type. To our knowledge, this is the first report describing the characteristic features of BCC among Egyptians.

## Introduction

Basal cell carcinoma (BCC) is the most frequently diagnosed malignancy with increasing annual incidence. Also, it is the most common malignant epithelial tumor worldwide that statistically constituting 80% of keratinocyte cancers. This malignant epithelial neoplasm arises from the interfollicular basal cell layer of the epidermis, and it is mainly caused by chronic exposure to ultraviolet (UV) radiation of sunlight. Therefore, BCCs occur mostly in those areas of the body which are exposed to sun rays, particularly on the head and neck. Although BCC is a locally aggressive tumor with low metastatic activity, it is associated with comorbidity and increasing cost burden (Kim et al. 2019; Schreuder et al. 2022; Cameron et al. 2019; Tan et al. 2015, 2018).

BCCs are diagnosed by direct inspection and histopathological examination is necessary to ascertain the diagnosis and determine the risk of recurrence (Cameron et al. 2019; Roewert-Huber et al. 2007). BCCs are often histopathologically described as a proliferation of homogeneous, basaloid cells with a hyperchromatic nucleus and minimal, poorly defined cytoplasm that resemble the epidermal basal cells morphologically (Tan et al. 2018; Altamura et al. 2010).

Clinical types of BCC include nodular, superficial spreading, pigmented, morpheaform, and fibroepithelioma of Pinkus. Histopathological variants are nodular, micronodular, superficial, pigmented, infiltrative, morpheaform, metatypical, and fibroepithelioma. Less common types are keratotic, adenoid, clear cell, granular type, and BCC with sebaceous and eccrine differentiation. Basosquamous cell carcinoma is a rare subtype of BCC with areas of both basaloid and squamoid differentiation. Some consider basosquamous cell carcinoma and metatypical BCC as synonyms, whereas others are of the view that they are separate entities (Nedved et al. 2014).

The exact demographic properties and clinicopathological criteria for BCC in Egypt are not reported in the literature, so this work aims to report the recorded data for patients diagnosed pathologically with BCC during the last five years.

## Patients and methods

### Study design

A retrospective cross-sectional multicenter study.

### Inclusion criteria

Egyptian patients diagnosed pathologically with BCC in the period from January 2017 to December 2021.

### Exclusion criteria

Patients originating from geographical areas other than Egypt.

### Data collection

A collaboration of 4 dermatology centers of university hospitals with different geographical distributions [2 in Cairo (Al-Azhar, AFCM), 1 in North Egypt (Alexandria), and 1 in Upper Egypt (Sohag)]. The study was conducted in accordance with the Declaration of Helsinki and its amendments, and it was also approved by the Institutional Review Boards of all participating centers in the study. Written informed consents from the patients were obtained at the time of the biopsy.

We retrospectively reviewed the medical records and the pathology archives to obtain the baseline clinical and histopathological data needed for the study. Demographic data such as age, sex, occupation, sun exposure (the average number of hours spent outdoors every week), number of lesions, and Fitzpatrick skin type were collected for each patient and correlated to the studied parameters. The clinical classification of BCC subtypes was done using Rook's Textbook of Dermatology classification system (Madan and Lear 2016).

Histologic subtyping was performed according to the classification mentioned by Weedon including nodular, micronodular, cystic, superficial, pigmented, adenoid, infiltrating, sclerosing, keratotic, infundibulocystic, metatypical, basosquamous, fibroepitheliomatous, and mixed patterns (Weedon and Patterson 2015).

### Statistical analysis

The collected data were revised for completeness and accuracy, coded, entered, and analyzed using Statistical Program for Social Science (SPSS) version 26 (IBM, USA). Quantitative data were expressed as mean ± standard deviation (SD). Qualitative data were expressed as frequency and percentage. The suitable statistical tests were used according to the type of data.

## Results

A total of 544 patients with histopathologically proven BCCs were registered through the collaborated centers in this 5-year study. There was a slight male predominance with 326 patients (59.9%), while female patients were 218 (40.1%). Male to female ratio was about 1.5 to 1. The mean age of presentation was 61.6 ± 13.2 years with female patients showing younger age of onset.

**Fig. 1:**
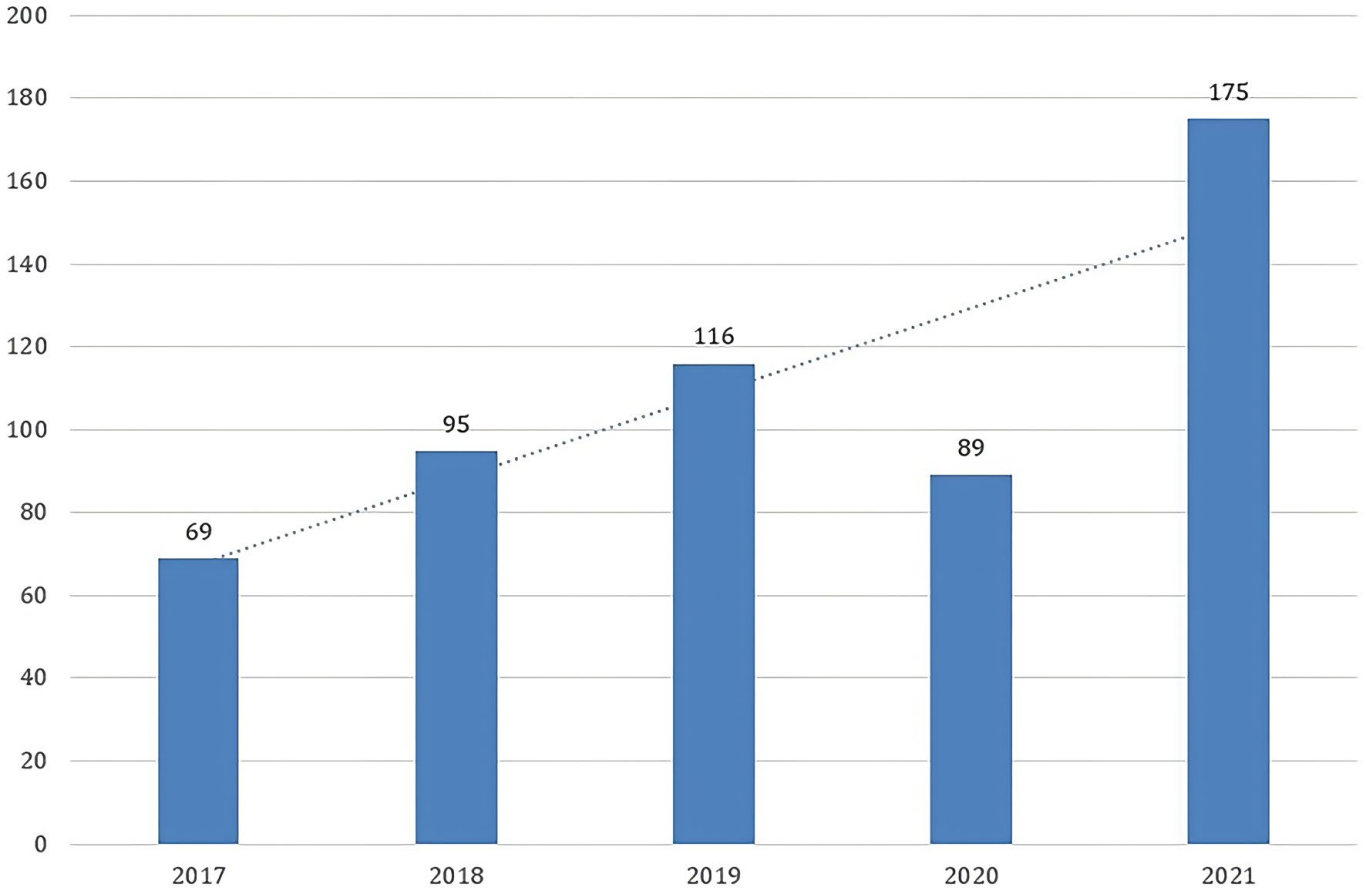
Number of annually diagnosed cases of BCC

Patients who reported a history of daily sun exposure (≥ 40 h spent outdoors per week) formed 71.3% (388 patients). The most common skin type affected was skin type III in 316 patients (182 males and 134 females) as shown in Table 1. The results showed an increased incidence of BCC from 2017 to 2021 with a peak of incidence in 2021 as shown in Fig. 1.

**Table 1:** Epidemiological criteria of the studied patients

		Male (*N* = 326) (59.9%)	Female (*N* = 218) (40.1%)	Statistical test and *P* value	The whole studied patients (*N* = 544)
Mean age of presentation		63.9 ± 12.1	58.02 ± 14.1	**T = 5.11** ***P* < 0.001**	61.6 ± 13.2
Skin type	II	16 (4.9%) 182 (55.8%) 128 (39.3%)	8 (3.7%) 134 (61.5%) 76 (34.9%)	χ^2^ = 1.84 *P* = 0.398	24 (4.4%) 316 (58.1%) 204 (37.5%)
	III				
	IV				

T, independent sample *t*-test; χ^2^, chi-square test; *P* value < 0.001 is considered highly significant; *P* value > 0.05 is considered non-significant

**Fig. 2:**
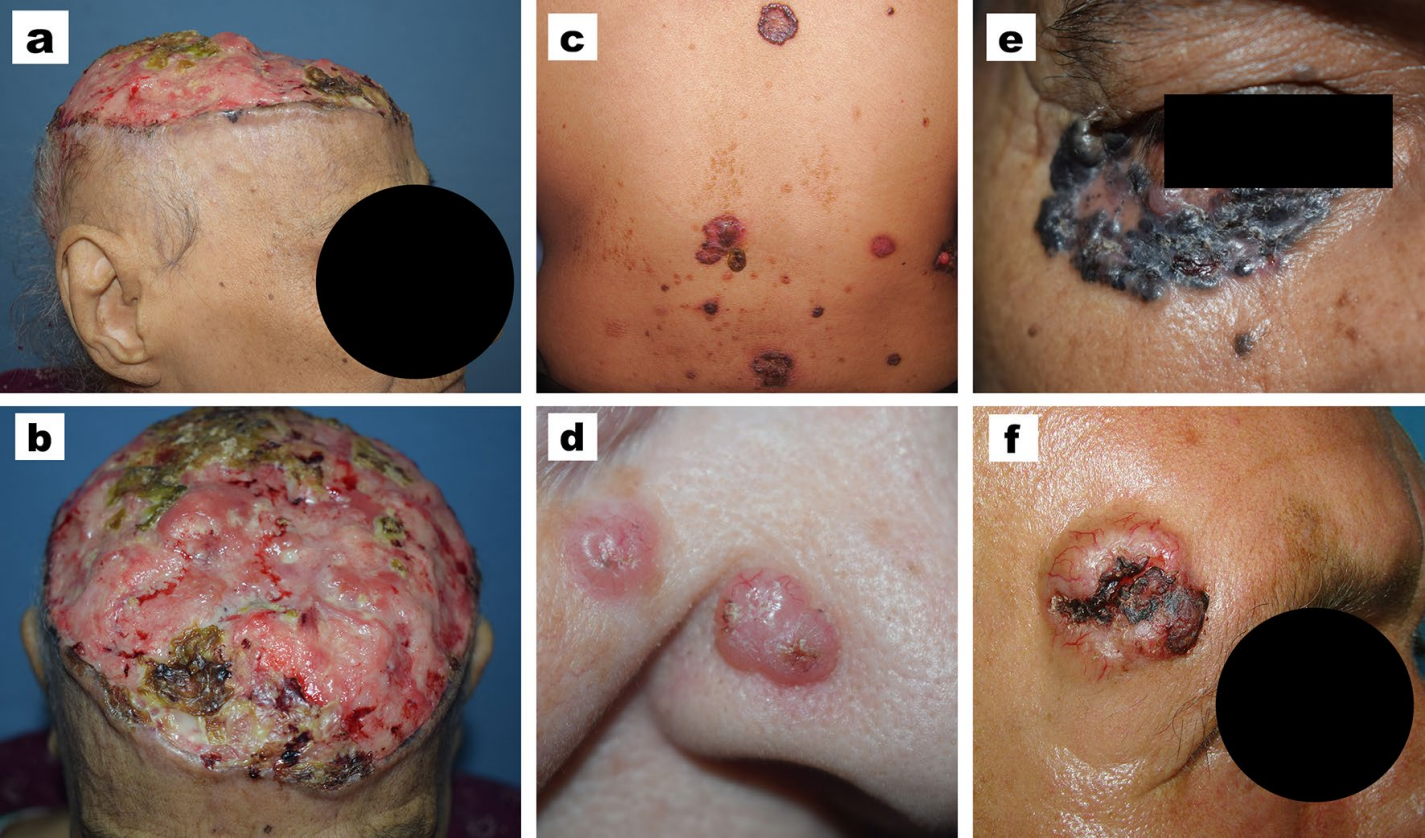
Different clinical presentations of BCC. **a**, **b** Giant BCC affecting the scalp with almost complete deroofing of the skin. **c** Multiple BCCs affecting the back. **d** Multiple BCCs affecting the nose and paranasal area. **e** Pigmented BCC affecting the right periorbital area. **f** Ulcerative BCC with marked telangiectasia located on the right temple region

Regarding the clinical criteria, most of the cases (93.8%) presented with solitary BCC, contrary to 34 patients who presented with multiple BCCs. It was observed that multiple BCCs were more prevalent in males. The head was the most commonly involved region (79.4%) where most of the lesions were located on the nose in 116 (21.3%) patients. Other sites of BCCs on the head were as follows: scalp in 96 (17.6%) patients, cheek 84 (15.4%), forehead 36 (6.6%), periorbital region 36 (6.6%), ear 15 (2.8%), post-auricular area 14 (2.6%), pre-auricular area 13 (2.4%), chin 12 (2.2%), and upper lip 10 (1.8%). About one third of cases (32.2%) had giant BCCs > 5 cm diameter (Table 2). The most common clinical variant, either males or females, was the ulcerative type followed by the non-pigmented nodular type (Fig. 2).

**Table 2:** Clinical criteria of the studied patients

		Male (*N* = 326) (59.9%)	Female (*N* = 218) (40.1%)	Statistical test and *P* value	The whole studied patients (*N* = 544)
Number	Single	300 (92%) 26 (8%)	210 (96.3%) 8 (3.7%)	**χ**^**2**^ **= 4.1** ***P* = 0.042**	510 (93.8%) 34 (6.2%)
	Multiple				
Site	Head	253 (77.6%) 12 (3.7%) 45 (13.8%) 16 (4.9%)	179 (82.1%) 7 (3.2%) 30 (13.8%) 2 (0.9%)	χ^2^ = 6.7 *P* = 0.081	432 (79.4%) 19 (3.5%) 75 (13.8%) 18 (3.3%)
	Neck				
	Trunk				
	Extremities				
Size	Non giant (≤ 5 Cm)	227 (69.6%) 99 (30.4%)	142 (65.1%) 76 (34.9%)	χ^2^ = 1.2 *P* = 0.271	369 (67.8%) 175 (32.2%)
	Giant (> 5 Cm)				
Clinical variant	Ulcerative	154 (47.2%)	90 (41.3%)	χ^2^ = 6.3 *P* = 0.095	244 (44.9%)
	Superficial	10 (3.1%)	8 (3.7%)		18 (3.3%)
	Nodular (non-pigmented)	101 (31%)	89 (40.8%)		190 (34.9%)
	Nodular (pigmented)	61 (18.7%)	31 (14.2%)		92 (16.9%)

χ^2^, chi-square test; NS, *P* value < 0.05 is considered significant; *P* value > 0.05 is considered non-significant

**Fig. 3:**
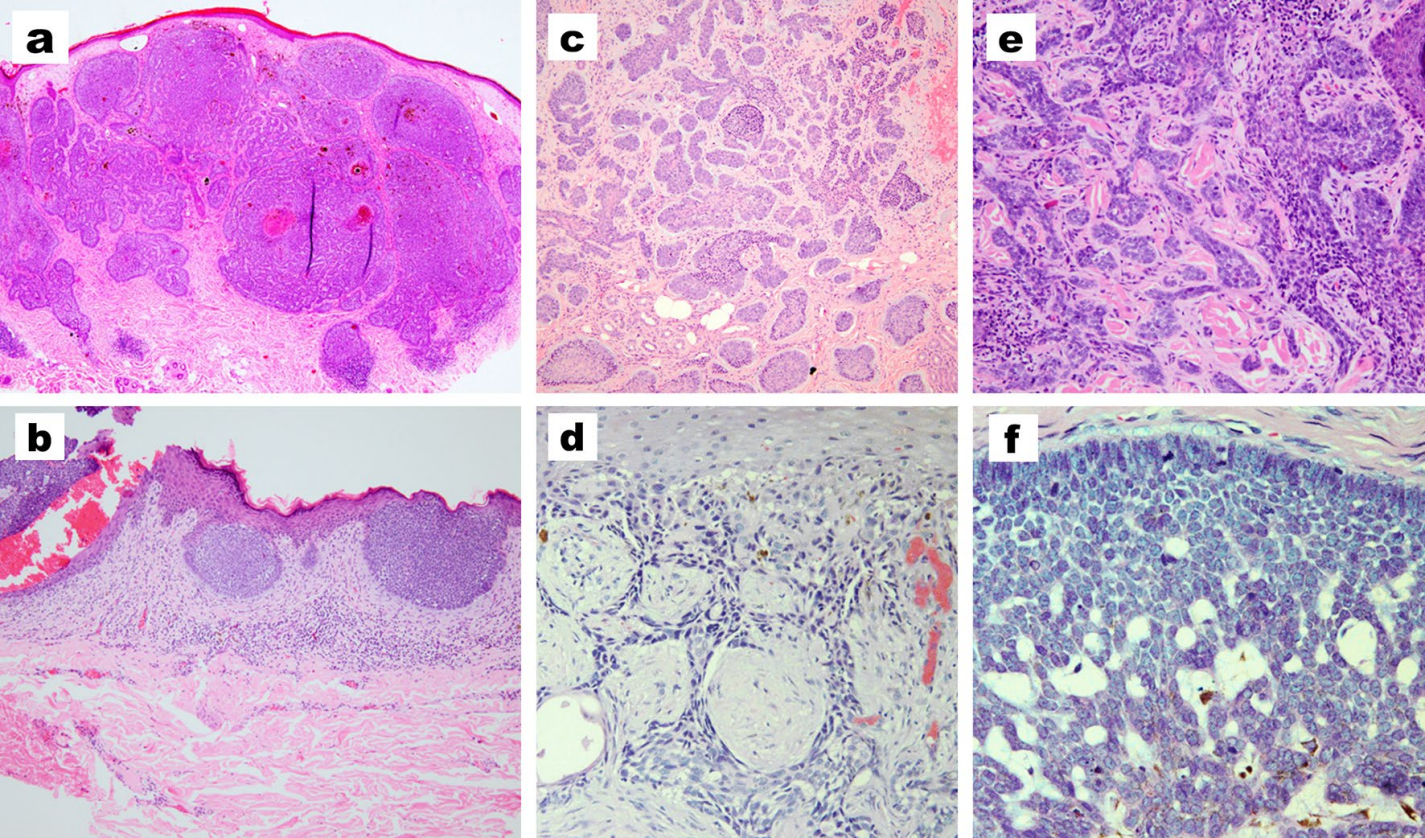
Different pathological types of BCC. **a** Nodular. **b** Superficial. **c** Micronodular. **d** Infiltrating. **e** Morpheaform. **f** Adenoid. (**a**, H&E × 100; **b**, **c**, H&E × 200; **d**-**f**, H&E × 400)

The mean duration of BCC was 3.9 ± 3.8 years, with no significant difference between males and females or giant and non-giant BCCs (Table 3). Regarding the pathological criteria, the nodular type was the most common variant followed by superficial BCCs (Table 4). There were no differences between giant or non-giant BCC regarding the pathological type (Table 5). Pigmented BCCs showed a slight likelihood of occurrence in male patients, while superficial BCCs were slightly more common than pigmented BCCs in female patients (Fig. 3).

**Table 3:** Duration of BCCs in the studied patients

	Duration in years	Statistical test and P value
The whole studied patients	3.9 ± 3.8	
Male	3.7 ± 3.5	MW = 34,280 *P* = 0.482
Female	4.2 ± 4.3	
Giant BCC	3.7 ± 3.5	MW = 31,071 *P* = 0.474
Non giant	4.07 ± 3.9	

*MW* Mann–Whitney *U* test; *P* value > 0.05 is considered non-significant

**Table 4:** Pathological criteria of the studied patients

		Male (*N* = 326) (59.9%)	Female (*N* = 218) (40.1%)	Statistical test and *P* value	The whole studied patients (*N* = 544)
Histopathological variants	Adenoid	20 (6.1%)	10 (4.6%)	**χ**^**2**^ **= 21.8** ***P* = 0.025**	30 (5.5%)
	Basosquamous	16 (4.9%)	6 (2.8%)		22 (4%)
	Fibroepithelioma of Pinkus	1 (0.3%)	0 (0%)		1 (0.2%)
	Keratotic	0 (0%)	1 (0.5%)		1 (0.2%)
	Infundibulocystic	1 (0.3%)	3 (1.4%)		4 (0.7%)
	Infiltrative	26 (8%)	18 (8.3%)		44 (8.1%)
	Micronodular	22 (6.7%)	12 (5.5%)		34 (6.3%)
	Mixed	10 (3.1%)	12 (5.5%)		22 (4%)
	Morpheaform	6 (1.8%)	4 (1.8%)		10 (1.8%)
	Nodular	150 (46%)	124 (56.9%)		274 (50.4%)
	Pigmented	38 (11.7%)	8 (3.7%)		46 (8.5%)
	Superficial	36 (11%)	20 (9.2%)		56 (10.3%)

χ^2^, chi-square test; NS, *P* value < 0.05 is considered significant

**Table 5:** Pathological criteria for giant and non-giant BCCs

		Giant (*N* = 175)	Non- giant (*N* = 369)	Statistical test and *P* value
Histopathological variants	Adenoid	11 (6.3%)	19 (5.1%)	**χ**^**2**^ **= 59.9** **P < 0.001**
	Basosquamous	14 (8%)	8 (2.2%)	
	Fibroepithelioma of Pinkus	0 (0%)	1 (0.3%)	
	Keratotic	0 (0%)	1 (0.3%)	
	Infundibulocystic	0 (0%)	4 (1.1%)	
	Infiltrative	2 (1.1%)	42 (11.4%)	
	Micronodular	19 (10.9%)	15 (4.1%)	
	Mixed	9 (5.1%)	13 (3.5%)	
	Morpheaform	2 (1.1%)	8 (2.2%)	
	Nodular	96 (54.9%)	178 (48.2%)	
	Pigmented	19 (10.9%)	27 (7.3%)	
	Superficial	3 (1.7%)	53 (14.4%)	

χ^2^, chi-square test; NS, *P* value < 0.001 is considered highly significant

**Fig. 4:**
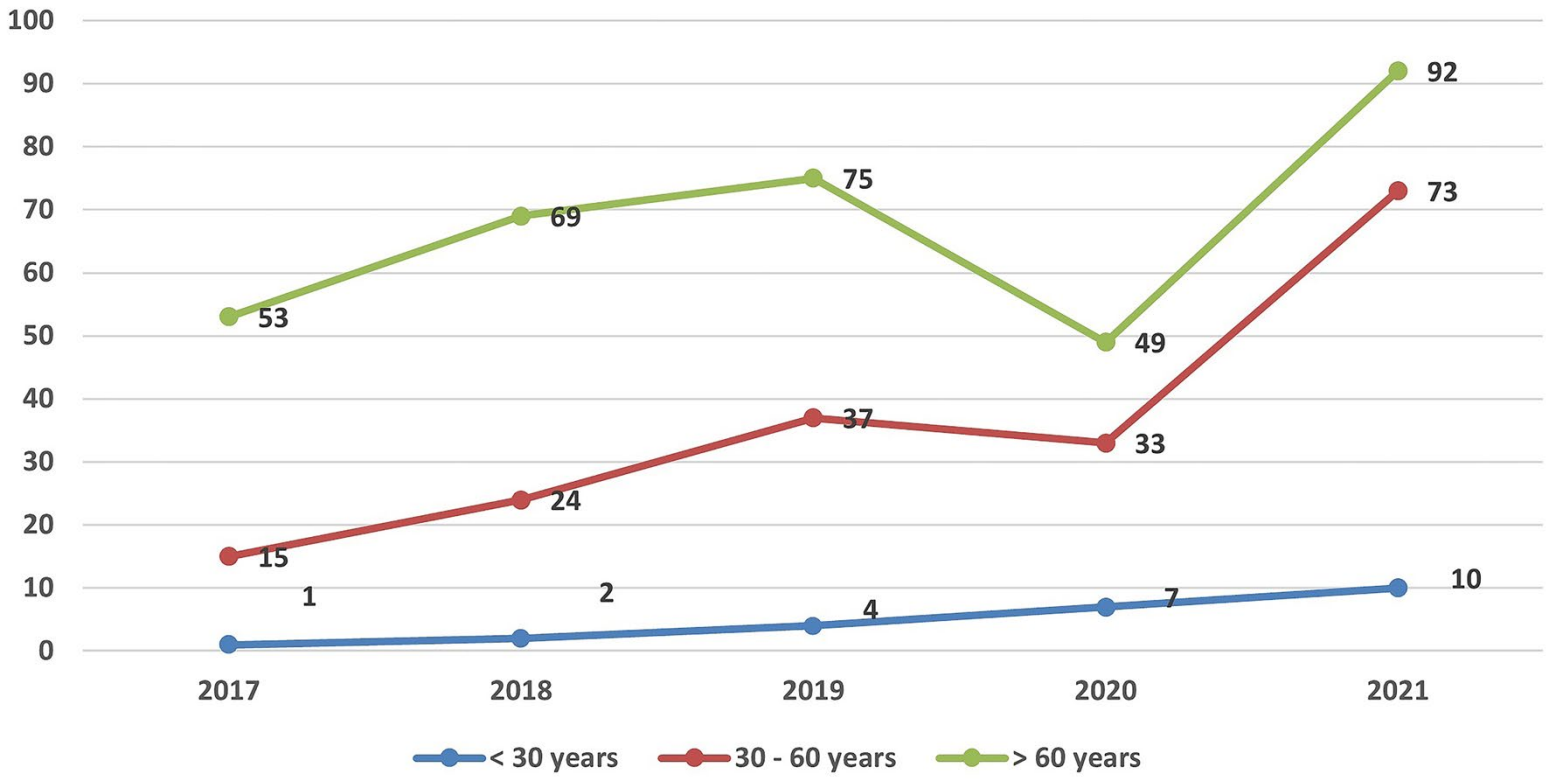
Number of diagnosed cases of BCC per year in relation to age

A noteworthy finding is the increasing number of diagnosed cases with BCC in patients under the age of 30 years (Fig. 4). Although it’s a non-significant increase, this is an alarming sign that BCC can affect younger age more commonly nowadays.

## Discussion

Basal cell carcinomas are common malignancies that usually show clear histomorphologic features, but in certain instances, they can display different patterns of differentiation leading to potential diagnostic confusion (Plaza et al. 2021).

The incidence of BCCs varies depending on race and on geographic factors such as latitude and sun exposure. The reported incidence rates per 100,000 person-years are highest in Australia (> 1000/100,000), around 100 in the UK, and 15–16.5 in Japan (Cameron et al. 2019; Lomas et al. 2012). However, the rates continue to increase worldwide. In the USA, the age-adjusted incidence rates almost doubled from the late 1980s to the middle 2000s in both men and women (Wu et al. 2013).

Pathobiologically, the majority of BCCs are caused by activation of the Hedgehog (HH) signaling pathway, which also serves as a therapeutic target. Following the discovery of a germline mutation in the patched homolog 1 (PTCH1) gene in basal cell nevus syndrome, mutations in genes associated with the HH signaling pathway, including PTCH1 and smoothened homolog (SMO), were discovered in sporadic BCCs (Tanese et al. 2018).

Multiple treatment modalities have been proposed for BCC. The surgical approach (including Mohs’ micrographic surgery) is the most widely accepted method, while other non-surgical methods may be tried (El-Khalawany et al. 2022).

### Epidemiological criteria

The results of this study showed that Egyptian patients with BCC showed slight predominance towards the male sex. These results are not different from what is reported in the literature as the male sex is a risk factor for BCC and the male to female ratio varied between 1.5–2 to 1 (Kim et al. 2019; Schreuder et al. 2022). This may be due to more frequent exposure of males to UV radiation which is related to the nature of their outdoor work.

The mean age of presentation in our cohort was 61.6 ± 13.2 which is consistent with what is reported in the literature that BCC is common in the 6th and 7th decades (Tan et al. 2015; Nedved et al. 2014; Madan and Lear 2016). However, it should be noted that in this cohort females showed slight earlier onset than males. Similar finding was observed by Chlebicka et al. (2021). This could be explained in western countries by the frequent use of tanning bath by females (Kappelin et al. 2022; Garcias-Ladaria et al. 2017), which is not a common habit for Egyptian females due to religious and social customs. However, a Turkish study reported that females showed earlier onset of BCCs, and they share the religious customs and ethnic race with Egyptians (Ozkanli et al. 2020).

Sun exposure is one of the most important risk factors for keratinocyte neoplasms (Kricker et al. 2017); this well explains that 71.3% of the studied cohort had a history of daily sun exposure. Studied patients with skin type III showed an insignificant increase rather than skin types II and IV. This could be explained by the fact that skin type III is more common in Egypt.

Apart from 2020, the total number of diagnosed cases in this study was steadily increasing. This could be explained by the increased awareness of patients towards the condition along with the advisory campaigns that were held for early detection of skin cancer. The drop in the number of diagnosed cases in 2020 could be owed to the restrictions made during the COVID-19 pandemic that asked people to stay at home and not to attend hospitals except for emergencies. Overall, there is an increase in the incidence of BCC cases. This coincides with the international trend of BCC incidence (Kim et al. 2019; Schreuder et al. 2022; Cameron et al. 2019).

### Clinical criteria

Solitary BCC was predominant in Egyptian patients which coincided with what is reported by Adachi et al. for Japanese patients with BCC (Adachi et al. 2018). A recent study from Netherlands showed that about 76–77% of patients had solitary BCC (Schreuder et al. 2022).

As male gender is considered one of the risk factors for BCC, this might explain that most of the studied patients with multiple BCCs were males (Cameron et al. 2019). Most of the cases presented were in the head region and the nose was the most commonly affected site, which agrees with what is reported in the literature denoting that the chronically damaged skin areas are usually involved with BCCs (Chlebicka et al. 2021; Cameron et al. 2019; Kricker et al. 2017; Kasumagic-Halilovic et al. 2019).

Vaca-Aguilera et al. (2019) reported that giant BCCs represent 1–2% of the whole BCCs. On the other hand, we reported a higher incidence (up to one third of cases) that was difficult to be explained either by the neglection of the tumor due to loss of significant symptoms or by the long duration of BCCs as our results showed no significant difference between giant and non-giant BCCs regarding the duration. The larger diameter of BCC in this study may owed to environmental, racial, or ethnical factors. Also, the mean duration in our cohort (3.9 years) was closely related to what is reported by Kumar et al. (2014) (4.7 years) and by Tan et al. (2015) (3.5 years).

Most of the previous reports described that the most clinical variant of BCC is the nodular type representing about 50%-80% of BCCs (Cameron et al. 2019; Nagarajan et al. 2020). In this study, about one third of cases were nodular non-ulcerative, and non-pigmented; while ulceration occurred in about half of lesions which is a higher proportion in comparison to the previous reports. Ozkanli et al. (2020) reported ulceration in 28.6% of BCCs cases, while Yap (2010) reported ulceration in 18% of cases. This difference should be considered, as it may indicate a more progressive course of the tumor in this racial type.

### Histopathological criteria

The most common pathological BCC variant in our cohort was the nodular type which is consistent with the data reported in the literature (Cameron et al. 2019; Tan et al. 2015; Ozkanli et al. 2020; Muzic et al. 2017). Regarding the giant BCC, Vaca-Aguilera et al. (2019) reported that infiltrating type was the most common pathological variant. However, in the present study, the nodular variant was the most common pathological variant of BCC in Egyptians regardless of the size of lesions.

The second most common pathological variant in our cohort was the superficial type which is similar to the reported data in the literature. Cameron et al. reported that the second most common pathological variant of BCCs was the superficial type with a relative increase in incidence in females (Cameron et al. 2019). In this study, pigmented BCCs showed slight probabilities of occurrence in males than the superficial type and this may be contributed to the ability of darker skin for tanning and colonization with tumoral dendritic melanocytes (Tan et al. 2015).

Unfortunately, we don’t have a national registry system for cases with BCC in Egypt. We hope that this study could open the eyes for the importance of such registry to exist in the near future.

## Conclusion

Our results showed that the annual incidence of BCC is increasing among Egyptians. UV radiation is considered a high-risk factor of BCC leading to a higher affection of the head region and more prevalence of the tumor in men. This study also reported some features of BCC in Egyptians such as the long duration of the tumor, the early onset in females, the higher percentage of giant types, and the predominance of nodular type. To our knowledge, this is the first report describing the epidemiological and clinicopathological features of BCC among Egyptians. We hope this registry would help in improving the prognostic outcomes of BCC among Egyptians.

## Data Availability

The data that support the findings of this study are available from the corresponding author upon reasonable request.
